# Wild food knowledge beyond documentation: what comes next?

**DOI:** 10.1186/s13002-026-00912-6

**Published:** 2026-05-28

**Authors:** Naji Sulaiman

**Affiliations:** University of Gastronomic Sciences, Piazza Vittorio Emanuele II 9, Pollenzo, 12042 Italy

The ethnobiology of wild foods has been largely shaped by a question that now appears increasingly insufficient: how much do people know? The field developed through the documentation of species, vernacular names, culinary preparations, gathering practices, and use categories, producing an extensive and genuinely valuable empirical record. Yet this tradition also rested upon an assumption that has rarely been examined directly. Ecological knowledge was largely approached as a cultural possession accumulated across generations and transmitted through language, memory, and practice, such that recording it before its disappearance became an urgent scholarly task. Documentation, therefore, became central to how the field defined its purpose and scholarly contribution.

The studies included in this collection suggest that this assumption is no longer fully adequate to the realities now shaping wild food systems across many parts of the world. Each contribution documents a specific ecological and cultural context. Across highly different regional settings, several recurring patterns become visible. Wild food knowledge persists most strongly where ecological engagement remains part of everyday life, labour, subsistence, and social practice. Where those conditions weaken, knowledge often retreats from practice into memory. Species may remain present within landscapes. Cultural identity may remain vibrant, and linguistic continuity may survive. However, the practical familiarity that once reproduced ecological knowledge through ordinary participation becomes increasingly fragile.

This distinction between biocultural memory and biocultural practice emerges repeatedly throughout the collection. It is also one of the most significant conceptual implications generated by the studies when considered collectively rather than separately. In central Tuscany, diachronic comparison across two decades demonstrates measurable contraction in the diversity of recognised and actively used wild food taxa despite continued cultural familiarity with many species. Informants frequently recalled plants and culinary uses associated with earlier periods of rural life, even where those practices no longer formed part of present-day subsistence or household routines. A similar trajectory appears in the coastal Mediterranean case studies from Gozo, Kasos, and Corsica, where ethnobotanical fieldwork increasingly records partial memories of foraging traditions detached from the everyday ecological interactions that once sustained them.

The studies conducted in the Italian Alps complicate this picture further. Minority languages among Cimbrian, Mòcheno, and Ladin communities remain institutionally recognised and culturally valued, while several forms of plant knowledge have nevertheless contracted substantially. At the same time, mushroom gathering and wild fruit foraging appear comparatively more resilient where communal land-use systems continue to maintain regular engagement with local landscapes. The contrast is important because it suggests that symbolic continuity alone does not necessarily sustain ecological practice. Knowledge appears more likely to persist where material and institutional conditions continue to support direct interaction with landscapes across generations.

Comparable dynamics emerge across very different geographical and cultural settings. In Kashmir, ecological familiarity remains unevenly distributed according to gender and occupational trajectory. Women engaged in gathering, preparation, and household provisioning retain deeper practical familiarity with wild plants and mushrooms than individuals whose educational and economic pathways have become increasingly detached from land-based livelihoods. In north-west Pakistan, displacement disrupted the social environments through which ecological knowledge had historically circulated between generations. The weakening of shared gatherings, collective movement through landscapes, and routine intergenerational interaction appears more consequential for transmission than the persistence or loss of cultural identity itself.

The same structural tendencies also appear in contexts where wild food systems remain highly active. In Guinea-Bissau, gathered plants continue to contribute directly to household food security and local economies, particularly during periods of agricultural shortage. In north-west Morocco, women vendors maintain ecological knowledge through local market systems that embed gathering within ordinary economic activity. Knowledge of seasonality, preparation, and plant identification continues to circulate because these practices remain economically meaningful and socially embedded. The study of edible insects in northern Uganda offers another important example. It suggests that entomophagy remains an ordinary and nutritionally recognised component of everyday food systems across multiple ethnic communities.

Across these cases, the studies point towards the same underlying dynamic. The continuity of ecological knowledge cannot be understood solely through cultural attachment, symbolic value, or the preservation of identity. The evidence assembled here repeatedly indicates that knowledge remains most resilient where gathering practices continue to be materially grounded in subsistence, labour, economic exchange, or everyday ecological engagement. The studies do not suggest that institutions alone determine the fate of ecological knowledge. Culture, memory, and identity clearly remain important. What repeatedly emerges across the collection, however, is that ecological knowledge is more likely to remain active where cultural meaning continues to be reinforced through regular practice and sustained engagement with landscapes.

This observation carries significant methodological implications for ethnobiology itself. Much of the field has relied upon synchronic documentation capable of recording what communities know at a particular moment in time. Such approaches remain indispensable, particularly in regions where knowledge has been poorly documented historically. Yet synchronic approaches also struggle to distinguish between actively practised ecological familiarity and knowledge retained through memory after everyday engagement with landscapes has weakened. The Tuscany comparison demonstrates the analytical value of diachronic research designs capable of tracing transformations across time using comparable methodologies. Without such approaches, ethnobiology risks documenting systems whose practical foundations may already be eroding beneath the surface of cultural continuity.

The Collection also highlights another issue that deserves greater attention within future research. The integrative review of Brazilian fruit trees and their pollinators reveals a substantial gap between ethnobotanical knowledge and ecological understanding. Of the hundreds of culturally important fruit species identified through ethnobotanical literature, only a minority possess documented pollination data. The ecological relationships sustaining many wild food resources therefore remain insufficiently understood despite extensive cultural documentation of their uses. Pollinator decline, habitat transformation, climatic shifts, and ecological fragmentation may alter the availability of culturally important species independently of whether ecological knowledge itself remains active within communities.

This ecological dimension expands the conceptual horizon of the field considerably. The continuity of wild food systems depends as much on ecological stability as on cultural and institutional continuity. In many regions, that ecological stability can no longer be assumed. An ethnobiology concerned only with documenting knowledge without integrating ecological process risks overlooking transformations occurring within the biological foundations of the systems it studies. The review from Brazil, alongside the ecological analysis conducted in north-west Yunnan, demonstrates the importance of approaches capable of bringing ethnobiology into closer dialogue with pollination ecology, environmental change research, and broader ecological sciences.

Several contributions also complicate simplistic narratives of decline by showing that continuity and transformation frequently coexist. The Swedish cloudberry study illustrates how a culturally significant gathering tradition may persist while its social organisation changes substantially. Commercial restructuring, labour shortages, imported seasonal workforces, and changing market conditions have altered who gathers cloudberries, under what conditions, and for whose benefit. The practice continues, but under very different economic and institutional arrangements from those that shaped earlier forms of gathering. Similar forms of restructuring appear across several studies in the collection, where gathered foods increasingly move between subsistence, market exchange, heritage discourse, gastronomy, and commercialisation.

Across the collection, continuity, adaptation, and erosion often occur simultaneously rather than as separate trajectories. Wild food knowledge may persist as active practice in some settings while contracting into memory in others. Ecological engagement may remain robust within economically grounded gathering systems while weakening in communities experiencing rapid socio-economic transition. Cultural identity may survive despite declining practical familiarity with landscapes. Ecological systems themselves may change independently of cultural continuity. These processes do not unfold uniformly, and the studies assembled here repeatedly caution against treating communities as homogeneous or static entities.

The implications for future research are substantial. Longitudinal and diachronic studies capable of tracing transformations over time deserve far greater attention within ethnobiology. Greater sensitivity to differences within communities, particularly across gender, labour roles, generation, and access to land, is equally necessary. Comparative work examining how institutional arrangements shape ecological engagement across different settings would also deepen understanding of why some systems remain operative while others fragment. At the same time, stronger integration between ethnobiology and ecological science will become increasingly important if the field is to understand how environmental change reshapes the material foundations upon which wild food systems depend.

Climate change, urban expansion, changing labour structures, migration, commercialisation, and transformations in land use are already reshaping relationships between communities and landscapes across many of the regions documented in this collection. Ethnobiology now faces questions that extend beyond the documentation of ecological knowledge alone. The field increasingly confronts questions concerning the conditions under which ecological practice remains possible, how those conditions change, and what forms of continuity or transformation emerge as a result.

Wild food knowledge may persist in archives, festivals, markets, and forms of cultural memory long after the practices that once sustained it have weakened. Such continuity remains culturally significant, yet it does not necessarily indicate the persistence of ecological practice itself. Ethnobiology risks misunderstanding this distinction when documentation becomes detached from the social, material, and ecological conditions through which knowledge is actively reproduced.

The studies gathered in this Collection show that wild foods continue to contribute to nutrition, livelihoods, identity, and everyday ecological familiarity across diverse regions of the world. They also indicate that the continuity of these systems depends upon the persistence of the social, material, and ecological conditions that sustain regular engagement with landscapes. The central challenge facing ethnobiology is becoming increasingly clear: documenting what communities know about wild foods is no longer sufficient without understanding the conditions that allow such knowledge to remain active, practised, and ecologically grounded.

